# A teleofunctional account of evolutionary mismatch

**DOI:** 10.1007/s10539-016-9527-1

**Published:** 2016-05-06

**Authors:** Nathan Cofnas

**Affiliations:** Department of History and Philosophy of Science, University of Cambridge, Free School Lane, Cambridge, CB2 3RH UK

**Keywords:** Evolutionary mismatch, Evolutionary novelty, Environment of evolutionary adaptedness, Evolution of intelligence, Proper function, Teleosemantics

## Abstract

When the environment in which an organism lives deviates in some essential way from that to which it is adapted, this is described as “evolutionary mismatch,” or “evolutionary novelty.” The notion of mismatch plays an important role, explicitly or implicitly, in evolution-informed cognitive psychology, clinical psychology, and medicine. The evolutionary novelty of our contemporary environment is thought to have significant implications for our health and well-being. However, scientists have generally been working without a clear definition of mismatch. This paper defines mismatch as *deviations in the environment that render biological traits unable, or impaired in their ability, to produce their selected effects* (i.e., to perform their proper functions in Neander’s sense). The machinery developed by Millikan in connection with her account of proper function, and with her related teleosemantic account of representation, is used to identify four major types, and several subtypes, of evolutionary mismatch. While the taxonomy offered here does not in itself resolve any scientific debates, the hope is that it can be used to better formulate empirical hypotheses concerning the effects of mismatch. To illustrate, it is used to show that the controversial hypothesis that general intelligence evolved as an adaptation to handle evolutionary novelty can, contra some critics, be formulated in a conceptually coherent way.

## Introduction

When the environment in which an organism lives deviates in some essential-but-not-yet-defined way from that to which it is adapted, this is described as “evolutionary mismatch,” i.e., between the ancestral and the current environment (Confer et al. 2010; Lloyd et al. 2014). Elements of the current environment that deviate from the ancestral one are described as “evolutionarily novel” (Irons 1998; Sterelny 2010).^1^ Those that do not, as “evolutionarily familiar” (Kanazawa 2004a). The environment(s) to which an organism is adapted can be termed its ancestral environment (AE).

The notion of mismatch plays an important role, explicitly or implicitly, in evolution-informed cognitive psychology (e.g., Confer et al. 2010; Tooby and Cosmides 1990), clinical psychology (e.g., Gilbert and Bailey 2000; Nesse 2004), and medicine (e.g., Eaton et al. 1988; Williams and Nesse 1991). However, scientists have generally been working without a clear definition of mismatch. This paper defines mismatch as *deviations in the environment that render biological traits unable, or impaired in their ability, to produce their selected effects* (i.e., to perform their proper functions in Neander’s 1991a, b sense). The machinery developed by Millikan in connection with her account of proper function, and with her related teleosemantic account of representation, is used to identify four major types, and several subtypes, of evolutionary mismatch.

Defining mismatch (or evolutionary novelty) in this way leaves it an open question whether different types of evolutionary novelty are good or bad for either the fitness or the general welfare of organisms. Many evolutionarily novel aspects of our contemporary environment are thought to have negative implications for our health and well-being (see Barrett 2007, 2010; Buss 2000; Lloyd et al. 2014: 4; Nesse 2004; Williams and Nesse 1991: 13–16). However, evolutionarily novel technology and systems of social organization have allowed our species to greatly increase its numbers (adaptedness) and have removed many evolutionarily *familiar* sources of suffering. Not all deviations from ancestral conditions are bad, and the neutral definition of mismatch defended in this paper captures that fact.

### A previous definition of mismatch

In one of the few previous philosophical analyses of the concept, Lloyd et al. (2014: 4) define mismatch as “*a [detrimental] consequence that results from a trait that evolved in one environment being placed in another environment*.” “‘Detrimental,’” they say, “will usually be defined in terms of evolutionary fitness, but it can also refer to welfare in more general terms” (6).

This rules out by definition the possibility of mismatch being beneficial. As Lloyd et al. say: “Traits that evolved in one environment need not be detrimental in a second environment; they can be neutral or fortuitously beneficial, but these cases are excluded by the term ‘mismatch’, which restricts our attention to the detrimental cases” (5). We need to define mismatch in more neutral terms if we wish to investigate how deviations from the AE can be bad *or* good. Take an example of mismatch considered by Lloyd et al.: A person who is able to tan and “who spends a lot of time indoors or covered with clothing will experience sunburn when their skin is suddenly exposed to the sun, a pattern of variation that seldom, if ever, occurred in their” AE (14). Clearly, sunburns per se are bad from both a fitness (they increase the risk of cancer) and a general-welfare perspective. But the evolutionarily novel situation of spending a lot of time indoors and/or wearing clothing, though it may increase the risk of sunburn in some individuals, may *on the whole* be a good thing—both fitness- *and* welfare-wise. Deviations from AE conditions can have negative, positive, or mixed effects on fitness and welfare. Furthermore, in actual scientific debates in which the consequences of evolutionary novelty are at issue, it is not adequate to define evolutionary novelty as having only negative consequences. One of these debates will be discussed in the following section.

## The scientific use of an account of evolutionary mismatch/novelty: the evolution of general intelligence

Some intelligence researchers hold that general intelligence (*g*) is an adaptation for dealing with evolutionary novelty (Chiappe and MacDonald 2005; Kanazawa 2004a). Byrne (1995: 38) argues that intelligence may evolve as an adaptation “in generalist animals…to exploit continually changing environments, since they must daily cope with novelty in order to survive.” Sterelny (2012: 3) sees the challenge posed by “evolutionarily novel features of the environment” as having been an important driver of hominin cognitive evolution. Critics raise two objections based on ambiguity in the concept of evolutionary novelty.

First, they say that the proposition that something is an adaptation for “evolutionary novelty” is logically contradictory: Adaptations can only evolve in response to *recurrent* features of the environment. If evolutionary novelty is understood as *non*recurrent features of the environment, it is impossible to evolve an adaptation to deal with it (Penke et al. 2011).

Second, they say that “the division between ‘evolutionarily familiar’ and ‘evolutionarily novel’ is so vague that one could legitimately argue that the same phenomenon was both ‘evolutionarily familiar’ and ‘evolutionarily novel’” (Dutton 2013: 612). Kanazawa (2010)—who advocates the hypothesis—claims that vegetarianism and liberalism are evolutionarily novel. From one perspective these things surely are evolutionarily novel: Until recently all our ancestors ate meat and did not go out of their way to help distant, unrelated people (what Kanazawa sees as the goal of liberalism). But with a little ingenuity we could think up reasons for regarding vegetarianism and liberalism as evolutionarily familiar. Dietary taboos are a human universal, and vegetarians can be regarded as members of a moral tribe; liberalism can be interpreted as an extension of the cooperative ideology that prevailed among hunter–gatherers. The general-intelligence-as-an-adaptation-to-evolutionary-novelty hypothesis will not yield any testable predictions if it is unclear what features of the environment are novel or familiar.

Notice that Lloyd et al.’s (2014) definition of mismatch as *harmful deviations from the AE* cannot be used to resolve this debate. The contention is that general intelligence evolved to handle novelty, which includes taking advantage of new *opportunities*. Organized social codes might be evolutionarily novel, and we might have evolved greater intelligence in order to deal with them, but the development of social codes presumably was positive for our species.

The definition of evolutionary novelty defended here could potentially be used to answer both of the above criticisms. First, it gives sense to the idea of *recurrent* evolutionary novelty (Byrne 1995; Kanazawa 2004a; Sterelny 2010). Sterelny (2010: 59) attributes our ability to “negotiate evolutionarily novel problems” in part to the fact that “novelty is not new” for our species. “Our lineage has repeatedly experienced demanding environmental change.” Byrne (1995: 38)—as quoted above—refers to “continually changing environments” that repeatedly confront animals with “novelty.” There is a conceptually coherent sense in which the environment can be recurrently novel over evolutionary time. The environment can remain evolutionarily novel indefinitely, as long as it continually changes so as to disrupt the performance of a species’ proper functions. (This idea seems implicit in the aforecited works.) In regard to the second criticism, the definition of evolutionary novelty given here can, of course, resolve the tricky cases, such as those mentioned above or discussed in Dutton (2013). Analysis will likely reveal that in some cases a feature of the environment may be partly evolutionarily familiar and partly novel.

The debate about the evolution of intelligence illustrates that “mismatch” may be more than a verbal category that is useful for scientists. It may potentially be something out there in the world that has actual consequences for evolution. Whether this is the case is an empirical, not a philosophical, question. But empirical hypotheses concerning the effects of mismatch require a coherent concept of mismatch in order to be formulated. The philosophical analysis given here attempts to clarify the concept.

## Groundwork for cataloging the types of evolutionary novelty

### Definition of evolutionary novelty

Neander (1991a, b) defines the “proper function(s)” of a biological trait as the effect(s) of the trait that it was selected *for*—the effects that played a causal role in its selection. She terms these the *selected effects*. Evolutionary familiarity is defined here as the environmental conditions necessary for biological traits to perform their (Neanderian) proper functions, i.e., to produce the effects that are responsible for their *recent* (Godfrey-Smith 1994) evolution and proliferation and/or maintenance. Evolutionary novelty is defined as deviations in the environment that prevent biological traits from performing, or impair their ability to perform, such functions. Such impairment is not necessarily bad for organisms from the standpoint of either fitness or welfare. Mice may possess mechanisms the proper function of which is to escape from cats. These mechanisms do not perform these functions in cat-free environments, but cat-free environments are good for mice. In humans, it is thought that depression is an adaptation for dealing with certain types of situations, causing the depressed person to withdraw from hopeless goals (Nesse 2000, 2004). Nevertheless, an environment that never triggered depression would be a good one (ceteris paribus; cf. Garson 2015: 177).

### Millikanian proper functions

In Millikan’s (1984) system, a *direct* proper function of *x* is *F* if the reason that *x* exists with character *C* is because, by virtue of *C*, *x* can perform *F* (26). For something to have a direct proper function, it must be a member of what she terms a “reproductively established family.” Different items belong to the same reproductively established family when they resemble each other due to later items being produced by copying, or reproduction of, earlier ones (or “models”; 18–19). The properties by virtue of which one item is a reproduction of another are “reproductively established properties” (20).

The proper function of a mechanism is *relational* when it is the function of the mechanism “to do or to produce something that bears a specific relation to something else” (39). For example, Smith’s dwarf chameleon (*Bradypodion taeniabronchum*) camouflages itself by changing color. The “pigment-rearranging device” of chameleons has the relational proper function to bring the color of the chameleon in a certain relation—namely, “same color as”—to the surface upon which it is sitting (39).

When a mechanism with a relational proper function is faced with the type of entity toward which it is its function to bring about a certain relation, the mechanism acquires an *adapted proper function*: the function to produce the specific item or situation that bears the appropriate relation to the given entity in this particular instance (40). As noted, the chameleon’s pigment-rearranging mechanism has as a relational proper function to make the chameleon’s color the “same color as” the surface upon which the animal finds itself. If the color of the surface is specified as, say, *brown*, then the pigment-rearranging mechanism has as its *adapted proper function* to turn the chameleon *brown*. When a device performs its adapted proper function, it is termed an *adapted device*. The entity to which the device is adapted is its *adaptor* (40).

The proper functions of adapted devices correspond to the proper functions of their producing devices that “lie *beyond* the production of these adapted devices themselves” (41). The proper functions of adapted devices are termed *derived proper functions* (41), and are of two types. *Adapted* derived proper functions are fixed by the device-producing mechanism and its particular adaptor. *Invariant* derived proper functions of an adapted device correspond to what can be described as the ultimate purpose of the producer. A *particular* bee dance—the adaptor of which is a source of nectar sighted by the dancing bee—has as its adapted derived proper function to induce the dancer’s sisters to fly a certain distance in a specific direction (say, 100 meters north). Its invariant derived proper function is to guide them to nectar.

### Proper functions and evolutionary mismatch

Again, this paper defines evolutionary mismatch/novelty as deviations in the environment that prevent biological traits from producing, or impair their ability to produce, their selected effects (what Neander defines as “proper function”). The taxonomy of evolutionary mismatch that will be given here is generated by the following observation: The property of being “a (Neanderian) proper function” is *logically coextensive* with that of being “either a (Millikanian) direct *or* invariant derived proper function of a biological trait.” If a trait *x* has been selected for having character *C* because, by virtue of *C*, *x* can perform *F*, *F* is the direct proper function of *x*, and also its *selected effect*—it has been selected because it does *F*. If a trait *y* has been selected because it produces adapted devices that bring about certain relationships to things in the world, the *invariant derived proper function* of the adapted device is the effect for the sake of which the mechanism that produced it was selected—invariant derived proper functions are selected effects.

To elaborate on the last point, traits with relational proper functions—which produce adapted devices with derived proper functions—can be favored by natural selection when they tend to bring about certain *invariant* results in the AE: “getting honey,” “escaping lions,” “catching mice,” and the like. It is these final, invariant results which are the *ultimate* evolutionary purposes of such traits—their *selected* effects. Obtaining them is the invariant derived proper function of adapted devices. If environmental conditions change such that traits with relational proper functions tend to produce adapted devices with very different *adapted* derived proper functions (e.g., flying 100 meters *south* rather than *north*), while continuing to fulfill *invariant* derived proper functions (e.g., getting honey), this change is (ceteris paribus) irrelevant from an evolutionary perspective: The trait continues to produce the effects for which it was selected. That is why only environmental change that prevents an adapted device from fulfilling, or impairs its ability to fulfill, its *invariant* derived proper functions is “evolutionarily novel” according to the definition given here.

### Devices with relational proper functions must consume *representations* of states of the environment

Devices with relational proper functions (which produce adapted devices with invariant derived proper functions) “do or…produce something that bears a specific relation to something else” (Millikan 1984: 39). The entity/world state toward which the mechanism has the function of bringing about a certain relation is called its *adaptor*. Any biological structure with a relational proper function performs its adapted proper function (in the normal way) in response to correct representations of the environment, specifically to the adaptor.

To perform relational proper functions, organisms must evolve some way to track the relevant environmental conditions. For an organism to track some environmental condition *E*, the instantiation of *E* must increase the probability of some type of state *M*_*P*_ (*p* for “producer”) occurring in the organism. When *M*_*P*_ is tokened, the mechanism *M*_*C*_ (*c* for “consumer”) in the organism that uses *M*_*P*_ as a sign that *E* is instantiated can exhibit a response appropriate for *E*. When *M*_*P*_ is in fact caused by *E*, *M*_*C*_ performs its proper function in exhibiting the response appropriate to *E* upon the registration of *M*_*P*_. According to Millikan (1989), *M*_*P*_ tokens *represent* the conditions that are necessary for the mechanism that consumes *M*_*P*_—namely, some mechanism *M*_*C*_—to fulfill its proper function.

To illustrate with camouflaging in Smith’s dwarf chameleon, the chameleon has to have some way of tracking the color it is sitting on. To do this, it must use some sort of inner intentional icons to represent different colors. There is no consuming mechanism that uses the skin color as a sign of anything, so the skin itself has no representational content (Millikan 1984: 118; 1989: 283). But in order for the pigment-arranging mechanism to perform its relational proper function (in the normal way) on (e.g.) a brown surface, a chameleon must have some state—an inner intentional icon—that covaries with and represents “brown surface.”^2^

### Four types of evolutionary mismatch/novelty

In Millikanian terms, evolutionary mismatch is defined as deviations in the environment that render biological traits unable, or impaired in their ability, to perform their direct proper functions *or* invariant derived proper functions. Biological traits perform their direct proper functions when the trait itself develops (unimpaired), and environmental conditions are such that the trait produces the effect for which it was selected. Environmental change that prevents traits with direct proper functions from developing, or that prevents fully developed traits with direct proper functions from producing their selected effects, constitutes two types of mismatch labeled Un(der)development-inducing and Ineffectiveness-inducing, respectively. Biological traits with relational proper functions perform their invariant derived proper functions when the organism correctly represents the environment (namely, the adaptor), and the adapted device it produces in response to that representation brings about the right adaptive relationship vis-à-vis the adaptor. Environmental change that causes the organism to misrepresent, or to fail to represent, the environment constitutes a third type of mismatch labeled Misrepresentation-inducing. If the environment changes so that the ways in which the organism *uses* representations fail to bring about the right invariant end results, this constitutes a fourth type of mismatch labeled Misresponse-inducing.

The four types of evolutionary mismatch—the four ways in which traits can fail to produce their selected effects—are discussed in turn in the following four sections.

## Type 1—Un(der)development-inducing: failure of a trait with a direct proper function to develop

The environment may change so that a biological trait that has a direct proper function simply fails to develop, or is impaired in its development. For example, human arms and hands have the direct proper function to be used for grasping (cf. Neander 1991a: 174). However, if a person develops in a womb that contains thalidomide, these structures may be greatly impaired in their development. Thalidomide in the womb is evolutionarily novel because it prevents biological traits (i.e., arms and hands) with direct proper functions from developing, or from developing well enough to perform their direct proper functions (i.e., grasping). A less dramatic example among humans is learning to read as a child, which leads to myopia among genetically susceptible individuals (Williams and Nesse 1991: 16).

## Type 2—Ineffectiveness-inducing: failure of a trait to perform its direct proper function despite unimpaired development

If a biological trait has a direct proper function, the environment may change so that, although the trait physically develops unimpaired, it fails to perform its proper function. Prior to the industrial revolution, peppered moths (*Biston betularia*) in England were nearly all of the “*typica*” form—white with black spots. According to the well-known story of industrial melanism, this coloration camouflaged the moths against lichen-covered trees. In the mid-1800s, pollution from factories killed the lichens, exposing the dark tree bark. This environmental change rendered the moths’ camouflaged structure unable to perform its direct proper function, although the moths continued to develop unimpaired (Cook et al. 2012).

### Learning

Millikan (1984: 24–25) identifies two ways of learning: An organism may repeat behavior that was previously “rewarded” (or met some other set of criteria), or it may imitate behavior that it observed in another organism. In the former case, the direct proper function of the learned behavior is to obtain the reward. In the latter case, it is to perform the function that the learner associated—by observation—with the behavior in question (28). In either case, a learned behavior may be as bizarre or as novel as you wish, but it seems that the conditions that produced it cannot be evolutionarily novel, according to the definition given here, as long as the reward is obtained or the (observed) function is performed. For conditions to be evolutionarily novel, some trait has to fail to perform its direct (or invariant derived) proper function as a result of environmental change. How can successfully performed learned behaviors reflect evolutionary novelty?

The answer is that the biological mechanisms that *underlie learning* perform their direct proper functions only when they produce (learned) behaviors with particular characteristics—namely, those characteristics by virtue of which the behaviors performed certain functions in the AE, which caused the (past) proliferation of (tokens of) the underlying learning mechanism (cf. Millikan 1989: 292). Learning mechanisms fail to perform their direct proper functions when they do not produce learned behaviors with such characteristics. For example, in their natural environment, cats learn to hunt when they are hungry. A housecat in an evolutionarily novel environment might learn that, whenever it shakes its owner’s hand, it gets a treat: It learns to shake hands when it is hungry. Being fed in response to performing this trick is evolutionarily novel vis-à-vis the cat’s mechanism responsible for learning how to obtain food. The mechanism has as its direct proper function to establish (by learning) a connection between hunting and eating. Under novel conditions, the mechanism causes the cat to obtain food in some other way, but not by performing its direct proper function.

## Type 3—Misrepresentation-inducing: misrepresenting or failing to represent the environment

In order to respond contingently to biologically important objects and situations, organisms may evolve traits with relational proper functions. In response to correct representations of their *adaptors*, these traits produce adapted devices which, under the right conditions, perform their invariant derived proper functions, thus accomplishing the ultimate goal for the sake of which the traits were selected. Environmental change that causes traits to fail to produce adapted devices that perform their invariant derived proper functions as a consequence of *misrepresenting* adaptors constitutes the third type of evolutionary mismatch. This section reviews the basic ways in which environmental change can cause organisms to misrepresent adaptors.

### Identifying objects

Under evolutionarily familiar conditions, organisms tend to correctly represent the objects in their environment as being of a biologically important type (such as *predator, prey, mother, sibling, potential mate*), though sometimes with a bias for false positives or false negatives (Godfrey-Smith 1991). Objects of certain types stimulate the production of certain states in the organism, which traits with relational proper functions use as representations of such objects.

The schema used to recognize object tokens of a particular type—i.e., to produce tokens of a state that the organism uses to track examples of that object type—can be acquired in either evolutionary or individual experience. Objects of different biologically important types have been—when encountered in evolutionary experience—more or less reliably associated with stimuli (or cues) of certain intensities in particular configurations (Eibl-Eibesfeldt 1989). The organism may evolve to use these cues in one of two ways. First, it may use them to pick out *examples* of an object type. The organism then *learns* to recognize tokens of the object type in question—forming a schema of it—through exposure to the examples. The cues used to recognize the original example(s) are not necessarily the same cues used to recognize the object after the schema has been formed on the basis of the examples. Second, the organism may, through genetic adaptation, possess innate sensitivity to the key stimuli (or configurational key stimuli) associated with biologically important object types. The following two subsections deal, respectively, with these two ways of forming object schemas.

#### Learning to recognize objects: imprinting

Organisms that rely on learning to form a schema of a biologically important type of object must be adapted to use (historically reliable) cues to guide them to correct examples (i.e., tokens) of the object type in question. When a schema formed in this way is incorrigible, the process is termed *imprinting* (Lorenz 1970: 124–133). The time in the organism’s life cycle in which imprinting occurs, or can occur, is termed the *critical period*.

If, during the critical period, any of the cues used by an organism to guide it to biologically correct examples of the object type to imprint on are removed, or are attached to tokens of other object types, this constitutes evolutionary novelty (cf. Garson 2015: 180–183). The result will be that the organism fails to imprint on any object, or imprints on an incorrect object type.

The *particular* object token(s) that an organism uses to form a schema of a biologically important type may, paradoxically, *not themselves be of that type*. Many birds and mammals use either their “parents” or their “siblings” to form schemas of “potential mates,” but *parents and siblings themselves are (usually) not potential mates*. A mallard drake (*Anas platyrhynchos*) raised by graylag goose (*Anser anser*) foster parents along with *mallard* siblings will flock with mallards but court *graylags*. Thus, it uses its siblings to form a schema of objects that elicit flocking behavior, and it uses its mother to form a schema of the objects that elicit courting behavior, although the mother *herself* will never be courted (Lorenz 1970: 280). A jackdaw (*Corvus monedula*) that has been reared by a human will, upon reaching sexual maturity, court *Homo sapiens*, but not the *particular* one who raised it (131). A Muscovy drake (*Cairina moschata*) raised by a human along with surrogate mallard siblings will, upon reaching sexual maturity, court mallards, indicating that it, unlike mallards and jackdaws, uses “siblings” as models to form the schema of “potential mates” (132).

For ducks and jackdaws, being raised by humans, or by or with birds of another species, is evolutionarily novel: Cues that, under evolutionarily familiar conditions, guide them to imprint sexual responses on examples of correct object types are, in the novel situation, attached to biologically incorrect objects. This causes them to misrepresent members of other species as potential mates and to produce an adapted device with the adapted derived proper function of courting these incorrect objects. Of course, this adapted device fails to perform its invariant derived proper function of bringing about copulation with (non-relative) conspecifics.

Under evolutionarily familiar conditions, humans imprint sexual *disgust* responses on close relatives. Recent research suggests that the sibling-inbreeding-avoidance mechanism in humans relies on two cues to establish siblinghood: childhood coresidence and maternal perinatal association (Lieberman et al. 2007). The latter cue refers to observing someone as an infant being cared for by one’s own biological mother—this cue is potentially available only to *older* siblings. The former cue is potentially available to older, younger, and same-aged siblings.

In humans, biological siblings who are not raised together often fail to imprint sexual disgust on each other. Contrary to thousands of years of popular belief—reflected most notably in the story of Oedipus—explicit knowledge *by itself* that someone is a relative appears to have little influence on one’s attitude toward sexual relations with them. In fact, it is very common for relatives (whether siblings or parents and children) separated at birth to feel, upon reunion, strongly attracted to each other, and even to act on these feelings. This phenomenon is termed “genetic sexual attraction” (Greenberg and Littlewood 1995). People who experience genetic sexual attraction, however, are typically averse to sexual relations with their adoptive family members. The situation of being raised without, and reunited in adulthood with, an opposite-sex sibling is evolutionarily novel: It greatly increases the likelihood of failure to represent opposite-sex siblings as *relatives* and thus to produce an adapted device that fulfills its invariant derived proper function of preventing sexual attraction to, and relations with, siblings.

#### Genetic adaptation to recognize objects

Any natural, evolutionarily familiar type of object will (in the AE) more or less reliably emanate stimuli in particular intensities and in particular configurations. Organisms can evolve to use a subset of these stimuli to recognize tokens of the object type. Registration of the “key” stimuli or configurational stimuli produces a token of a state type used by the organism to represent the object (Eibl-Eibesfeldt 1989: 55; Tinbergen 1951: 25, 27). An object is evolutionarily novel when it emanates key stimuli associated with an evolutionarily familiar object but in different intensities or combinations, *and/or* it emanates key stimuli associated with an evolutionarily familiar type of object but is *not* of the same biologically important type. Visual pornography, for example, consists of images/video emanating key stimuli associated with “sexually receptive mate,” and these key stimuli may be arranged in evolutionarily novel combinations. More examples will be given in the subsection on supernormal stimuli (below).

Consider also Dretske’s (1986) example of marine bacteria that swim toward oxygen-low water by means of magnetotaxis. These bacteria contain tiny magnets called magnetosomes. The magnetosomes of species in the northern hemisphere draw bacteria toward geomagnetic north, which is *downwards* and away from highly oxygenated surface water. Species in the southern hemisphere have their magnetosomes reversed: They are drawn toward geomagnetic south, which is downwards and away from surface water in that hemisphere. The magnetic pull is the key stimulus that the bacteria are genetically adapted to use to produce a representation of the direction of oxygen-low water.

The system that the bacterium uses to orient itself has a relational proper function. Its function is to bring about a certain relation between the organism and the oxygen-low water (namely, that the bacterium should swim toward and be in such water). “Oxygen-low water” is the adaptor—the thing toward which the system has the function of bringing about a relation. The bacterium has to represent the adaptor in order to respond to it. “Attraction-to-a-direction-at-a-time” (cf. Millikan 1989: 290) does this—it represents the direction of oxygen-low water. The adapted device produced in response to this representation^3^ is a specific orientation with the flagellum propelling the bacterium forward. The adapted derived proper function of the adapted device is to swim in the direction of the magnetic pull at that particular time. The invariant derived proper function is to swim in the direction of oxygen-low water.

Living in the southern hemisphere would be evolutionarily novel for a northern hemisphere bacterium and vice versa. In either case the orienting system in the bacterium would *misrepresent* its adaptor (low-oxygen water) as being upwards, because the key stimulus it uses to recognize oxygen-low water is associated with oxygen-high water. In response to the misrepresentation, the bacterium would produce an adapted device that fails to perform its invariant derived proper function.

### Identifying situations

Under evolutionarily familiar conditions, organisms tend to correctly represent the situations in their environment as being of a biologically important type, and, just as with objects, sometimes with a bias for false positives or false negatives. Tokens of biologically significant situation types stimulate the production of certain states in the organism, which devices with relational proper functions use as representations. Environmental deviations that cause situations to remain unrepresented, or to be incorrectly represented, constitute a subtype of evolutionary novelty.

#### Mismatch: misrepresenting situations

Consider social exchange (in our species). Rational choice theory often fails to predict peoples’ behavior in both artificial and natural social-exchange situations. About half of subjects cooperate in anonymous, one-shot prisoner’s dilemma games (PDGs), while rational choice theory predicts that they will defect (Kanazawa 2004b: 41). Kanazawa (2004b) hypothesizes that the more a social-exchange situation corresponds to situations that existed in the AE, the more likely people will be to behave according to the predictions of rational choice theory. Why do so many people cooperate when playing *anonymous, one*-*shot* PDGs when doing so amounts to giving money to an anonymous stranger? The answer, Kanazawa proposes, is that, in the AE, there was no such thing as anonymous social exchange, nor of guaranteed one-time interactions. The only way to interact with someone in the AE was face-to-face. And, unless you killed them, there was no such thing as interacting with someone and knowing that you would never see them again. Situations of social exchange activate specialized cognitive mechanisms, which do not take cues of “anonymity” or “one-time-ness” as input. In modern postindustrial society, anonymous and non-iterated social exchange is common—in some contexts, even the norm. Our behavior (e.g., cooperating in anonymous, one-shot PDGs and contributing to public goods) reflects a representation of our environment (namely, interactions are not anonymous or one shot) that is now often inaccurate. In anonymous, one-shot social exchange, our cognitive mechanisms produce an adapted device with the adapted derived proper function to be generous to our partner. The device fails to fulfill its invariant derived proper function of maximizing expected long-run payoff.

In response to cues reflecting specific environmental conditions, organisms can develop an adaptive phenotype by means of *developmental switches*. For example, some species of water flea (crustaceans of the genus *Daphnia*) develop hard shells if exposed to chemicals emitted by their predators. The shells are “metabolically costly,” but worth it if the water flea is in an environment with many predators (Garson 2015: 178–179). Garson defines “developmental mismatch” as occurring when a cue triggers the development of a phenotype that turns out not to be appropriate—for example, if the chemical associated with predators was emitted in an environment without any predators.

“Developmental mismatches” result from misrepresenting the environment. The developmental switch is part of a system with a relational proper function. The *adaptor* is the environmental situation (e.g., many predators), which must be correctly represented. The phenotype that develops is an adapted device with an invariant derived proper function to relate to the adaptor in the appropriate way. If the organism misrepresents the adaptor, it will develop a phenotype that fails to accomplish its invariant derived proper function.

### Supernormal stimuli

The releasing effect of key stimuli (used to identify either objects or situations) can be increased by intensifying their presentation (Tinbergen 1951: 44–46). Configurational key stimuli can also be intensified by exaggerating the stimulus relations in question (e.g., dolls, teddy bears, and certain cartoon characters; Eibl-Eibesfeldt 1989: 59–63). An object in which several key stimuli with additive releasing effects are combined may have a releasing effect greater than any, or almost any, natural object. That is, objects can be represented as being clear examples of a certain biologically important type, although they may not be of that type at all.

For practically all organisms, habitat selection is an important adaptive problem. Survival is closely tied to living in the right environment. Organisms evolve to be attracted to key stimuli associated with their biologically ideal environment, and to avoid environments that lack these stimuli. Humans seem to have evolved to prefer environmental conditions associated with the safest part of the Pleistocene African savannah: verdant, open spaces with scattered trees, especially in close proximity to clear water. When shown photographs of a variety of environments, children who have never been to a tropical savanna indicate that it would be the best environment to live in or to visit. Adults develop a comparable level of attraction to environments with which they are familiar, but continue to judge the savannah as appealing regardless of their individual experience (Balling and Falk 1982).

The idyllic savannah environment is recreated in an exaggerated form, albeit on a smaller scale, in the suburban lawn. The grass of the suburban lawn is greener, brighter, and more consistent than that which ever existed in the AE. Ironically, these lawns attain their supernormally stimulating effects due to the administration of health-threatening fertilizers and herbicides that sometimes reduce the fitness of those who come into contact with them (Barrett 2007: 67). The popular herbicide 2,4-dichlorophenoxyacetic acid, for example, threatens fitness in a dramatic way. Among men, exposure to this chemical is associated with reduced sperm quality; among pregnant women, with increased risk of miscarriage. Our attraction to key stimuli associated with the most fitness-promoting environment in the AE leads us to create an environment that needlessly threatens us, though we are compensated with a certain amount of aesthetic pleasure. Our representation of certain landscape features as indicating a vicinity of *maximum* safety is no longer accurate. Our habitat-selection mechanism produces an adapted device (namely, a desire to live in close proximity to supernormally stimulating lawns) that may fail to fulfill its invariant derived proper function (namely, leading us to an area that is more fitness promoting than the alternatives).

## Type 4—Misresponse-inducing: responding incorrectly to environmental conditions

The previous section dealt with the principles by which organisms produce states to track environmental conditions. This section deals with how traits with relational proper functions *use*, or *consume*, these states in order to bring about adaptive relationships vis-à-vis their adaptors.

Under evolutionarily familiar conditions, particular responses are elicited with certain *frequencies*, and are joined together to form *behavioral chains* to accomplish biologically important *goals* (i.e., invariant derived proper functions). When conditions change such that responses are elicited with different frequencies, or chains of behavior are not assembled correctly, or responses no longer achieve the biological purpose for which they evolved, the environment is evolutionarily novel.

### Behavioral chains

Biological goals are often accomplished by the successive performance of relational proper functions, each of which is released by a separate stimulus situation/adaptor and is subject to its own appetence (cf. Millikan 1984: 41).

In many cases, the completion of one relational proper function in a behavioral chain brings about the adaptor necessary to release the next relational proper function, and so on, until a biological goal is accomplished (through performance of the invariant derived proper function of the adapted device produced by the last relational proper function in the chain). This is particularly obvious in the case of reproductive behavior, which, in many animals, begins with courting (itself composed of a chain of behaviors), the performance of which brings about the stimulus situation/adaptor releasing copulation/insemination. This produces fertilized eggs or young, which in turn release parental behaviors.

Successful hunting behavior in cats (and many other predators) requires the integration of several separate motor patterns: stalking, pouncing, killing, and eating prey. Each of these behaviors can be released independently, and can be temporarily exhausted by repeated elicitation without reducing the appetence for the other behaviors that comprise the chain. Under natural conditions, successful stalking—released by the sight of a small animal, or signs of such an animal—brings about a situation that releases pouncing, which, when successful, leads to killing and finally to eating. Under natural conditions, cats learn to hunt when they are hungry. Under the evolutionarily novel conditions of apartment living, the elements of the hunting behavioral chain often become separated: The cat stalks shadows, pounces on balls of string, directs killing motions at random objects, and eats “cat food” from a dish.

### Frequency of elicitation of responses

Objects and situations for which a particular response is adaptive are encountered, or must be sought or brought about, with a particular *frequency* in the AE. Organisms typically evolve so that, under evolutionarily familiar conditions, particular responses are elicited by the right stimulus situations with the frequency appropriate to the AE. The drive to perform a behavior (a relational proper function) is typically matched to the appropriate frequency (Tinbergen 1951: 168). An environment is evolutionarily novel vis-à-vis an evolved response if it presents the eliciting stimulus situation (which is represented as the adaptor) with a greater or lesser frequency than it occurred in the organism’s evolutionary experience. In such environments, evolved responses may be elicited in biologically inappropriate situations (the organism produces an adapted device in response to the wrong adaptor) or fail to be elicited in appropriate situations (the organism fails to produce an adapted device in response to the right adaptor).

As noted in the previous subsection, the elements comprising a behavioral chain are elicited by different stimulus situations, and are subject to their own appetence. Such elements will often, under evolutionarily familiar conditions, be elicited with different frequencies. This occurs when performance of one element of the chain often does not produce the adaptor for the relational proper function that comes next in the chain. As discussed, hunting in cats involves the successive performance of four separate behaviors, viz., stalking, pouncing, killing, and eating prey. Under evolutionarily familiar conditions, the earlier elements of this behavioral chain are performed more often than the later, since the prey may escape at any point before it is successfully killed.^4^ Thus, cats possess drives to perform the later elements of this behavioral chain with successively lesser frequency. If a cat is locked in a room with live mice introduced in succession—under conditions where the mice may put up a fight but never escape—the cat responds in the beginning by performing the whole sequence of hunting behavior to completion. After it has eaten several mice, it will kill a few more without eating them. Then the cat will stalk and catch mice for a while, before letting them go. Finally, pouncing behavior is exhausted, and the cat will simply stalk mice for a while until this last appetence is exhausted (Lorenz 1966: 92–93).

## Summary and application to a scientific debate

This paper has delimited four types of evolutionary mismatch along with several subtypes (Table 1). This section seeks to illustrate the potential scientific use of this taxonomy by briefly considering its relevance for the hypothesis about the evolution of intelligence discussed earlier.

**Table 1:** Four types of evolutionary mismatch/novelty

Type	Subtype	Example
Type 1—Un(der)development-inducing: failure of a trait with a direct proper function to develop		Thalidomide in the womb prevents limbs from developing; reading from a young age causes myopia
Type 2—Ineffectiveness-inducing: failure of a trait to perform its direct proper function despite unimpaired development		*Typica* peppered moths in a polluted environment are not camouflaged
	Failure of a learning mechanism to produce the behavior for the sake of which it evolved	Apartment-living cats fail to learn to hunt when they are hungry
Type 3—Misrepresentation-inducing: misrepresenting or failing to represent the environment	Imprinting on the wrong type of object	Jackdaws raised by humans court *H. sapiens* upon reaching sexual maturity
	Failing to imprint	Siblings reared apart fail to imprint sexual disgust on each other (to avoid inbreeding)
	An object emanates key stimuli in different intensities or combinations from an evolutionarily familiar object	Supernormal stimuli (e.g., food sweetened with refined sugar); some visual pornography
	Key stimuli associated with a biologically important type of object are attached to an object of a different type	Visual pornography; teddy bears; artificially sweetened, sugar-free food; Facebook profiles
	Misrepresenting situations	Many people cooperate in anonymous, one-shot prisoner’s dilemma games; “developmental mismatch”
Type 4—Misresponse-inducing: responding incorrectly to environmental conditions	Behavioral chains are not integrated	The four main components of hunting behavior in apartment-living cats are not integrated
	Responses are elicited with different frequencies from those that occurred in evolutionary experience	Among most inhabitants of contemporary Western societies, genuine fear is elicited much less frequently than it was in the relatively violent and dangerous AE

As noted, some intelligence researchers argue that intelligence in humans, and potentially other animals, is an adaptation for dealing with evolutionary novelty (Byrne 1995: 38; Chiappe and MacDonald 2005; Kanazawa 2004a). These researchers have clearly been referring to Misrepresentation-inducing and Misresponse-inducing evolutionary novelty (Types 3 and 4).

If the cues used by a species to recognize biologically important objects and situations are rendered unreliable by environmental change (Type 3), and if no substitute cues are available, natural selection may put a huge premium on general reasoning ability: Only those organisms capable of interpreting unpredictable, transient evidence concerning the classification of objects and situations will be able to navigate the environment successfully. Consider a problem faced by a wide range of animals: distinguishing “friends” and “enemies.” Most social animals use a straightforward procedure to solve this problem, relying on key stimuli (e.g., the individual is/is not a member of my group). When social organization becomes more complex—as with coalition-forming chimpanzees—relying on simple cues to represent conspecifics as friends and enemies will not work. Among humans, enemies may emit every possible sign of friendship until the moment of treachery. And real friends sometimes act in confusing ways.

Seeing the conditions under which humans distinguish friends and enemies as evolutionarily novel helps to (partly) unify the social intelligence hypothesis and the hypothesis that intelligence evolved to deal with evolutionary novelty. Sterelny (2012: 6) argues that “[h]ominin cognitive evolution cannot have been driven mostly by external environmental change, as then we would expect similar trajectories in other species, and that we do not see.” But there may have been evolutionary novelty in early hominin’s social domain. Changes in social organization rendered their evolved mechanisms for identifying friends and enemies unable to perform their proper functions. Since there are no reliable cues that could be used to determine whether a would-be ally will remain faithful to you over the course of a long-term political campaign (such as existed in the AE), general reasoning ability is the only faculty that could help one determine whom to trust and to what extent.

## Conclusion

The method for cataloging evolutionary mismatch given here does not by itself resolve any scientific debates. But it may allow hypotheses concerning the effects of mismatch in Darwinian medicine (Williams and Nesse 1991), evolution-informed clinical psychology (Gilbert and Bailey 2000), and other fields to be formulated clearly. In regard to intelligence research, it could help make predictions about which species are likely to have undergone selection for general reasoning ability. What consequences the different types of mismatch have for species’ evolution and well-being are empirical questions—which can be investigated in the light of a clear mismatch concept.

## Footnotes

1 In biology, the term “evolutionary novelty” usually refers to novel phenotypes. Scholars of mismatch use the term to refer to novel environments.

2 Such representations are “pushmi-pullyu representations”: They are descriptive and directive at the same time (Millikan 1995).

3 Pushmi-pullyu representation—see note 2 (above).

4 Even killing movements are not always successful.
